# Insights into the cellular pathophysiology of familial hemophagocytic lymphohistiocytosis

**DOI:** 10.3389/fimmu.2023.1147603

**Published:** 2023-03-09

**Authors:** Erica A. Steen, Kim E. Nichols, Lauren K. Meyer

**Affiliations:** ^1^ University of California, San Diego, CA, United States; ^2^ Department of Oncology, St. Jude Children’s Research Hospital, Memphis, TN, United States; ^3^ Department of Pediatrics, University of Washington, Seattle, WA, United States

**Keywords:** hemophagocytic lymphohistiocytosis (HLH), cytotoxicity, hyperinflammation, cytokine, cytotoxic lymphocyte, natural killer cell

## Abstract

Familial hemophagocytic lymphohistiocytosis (fHLH) encompasses a group of rare inherited immune dysregulation disorders characterized by loss-of-function mutations in one of several genes involved in the assembly, exocytosis, and function of cytotoxic granules within CD8+ T cells and natural killer (NK) cells. The resulting defect in cytotoxicity allows these cells to be appropriately stimulated in response to an antigenic trigger, and also impairs their ability to effectively mediate and terminate the immune response. Consequently, there is sustained lymphocyte activation, resulting in the secretion of excessive amounts of pro-inflammatory cytokines that further activate other cells of the innate and adaptive immune systems. Together, these activated cells and pro-inflammatory cytokines mediate tissue damage that leads to multi-organ failure in the absence of treatment aimed at controlling hyperinflammation. In this article, we review these mechanisms of hyperinflammation in fHLH at the cellular level, focusing primarily on studies performed in murine models of fHLH that have provided insight into how defects in the lymphocyte cytotoxicity pathway mediate rampant and sustained immune dysregulation.

## 1 Introduction

Familial hemophagocytic lymphohistiocytosis (fHLH) is an inherited immune dysregulation disorder characterized by systemic hyperinflammation. In fHLH, excessive immune cell activation and the resulting cytokine storm drive the characteristic clinical features, which include fevers, multi-lineage cytopenias, organomegaly, and coagulopathy. Treatment aimed at controlling hyperinflammation and correcting the underlying immune defect is necessary to prevent progression to fatal multiorgan failure (1).

FHLH is caused by inherited loss-of-function (LOF) mutations in one of several genes required for cytotoxic lymphocytes (CLs), including CD8+ T cells and natural killer (NK) cells, to mediate target cell death. During a normal immune response, CLs recognize an antigen present on a target cell, resulting in the assembly of an immunologic synapse (IS) with that target cell. Through the coordinated activity of multiple intracellular proteins, lytic granules are trafficked to the site of target cell contact and released across the IS, triggering target cell death (2). In fHLH, defects along this cytotoxicity pathway impair the release or function of these lytic granules, preventing the induction of target cell apoptosis (3). While mutations in multiple genes have been shown to mediate an HLH phenotype (4), the most common genetic etiology of fHLH involves LOF mutations in *PRF1 (* 3 *)*, the gene encoding perforin. Perforin is contained within lytic granules and functions by forming pores in the target cell membrane, enabling proteases known as granzymes to enter the target cell and cleave protein substrates to rapidly induce apoptosis (5). Mutations in other genes involved in the cytotoxicity pathway, including *UNC13D, STX11*, and *STXBP2*, underlie other fHLH subtypes. These mutations impair the docking of lytic granules at the IS and their fusion with the plasma membrane, conferring varying degrees of cytotoxic dysfunction (6, 7).

At the cellular level, failure to eliminate target cells results in persistent CL activation. Activated CLs secrete pro-inflammatory cytokines that stimulate other cells of the immune system, which in turn secrete additional cytokines, resulting in a feed-forward loop driving sustained immune activation (8). Multiple studies have shown that CL activation, and particularly CD8+ T cell activation, is central to the pathophysiology of fHLH. One study demonstrated this using *Prf1^-/-^* mice which, when infected with lymphocytic choriomeningitis virus (LCMV), develop fatal hyperinflammation that recapitulates the clinical features of fHLH (9). Using cell depletion experiments, they demonstrated that mice with selective depletion of CD8+ T cells had prolonged survival following LCMV infection (10). Similarly, other studies have shown that adoptive transfer of cytotoxicity-competent CD8+ T cells into *Prf1^-/-^* (11, 12) or *Unc13d^-/-^* (13) hosts ameliorates disease, suggesting that CD8+ T cells are key drivers of immunopathology in fHLH.

Murine models have proven invaluable for advancing our understanding of the cellular processes by which defective cytotoxicity alters the behavior of CLs to mediate hyperactive immune responses. Specifically, models have been developed to study multiple subtypes of fHLH and related disorders, many of which recapitulate the clinical features of this disease following exposure to an infectious trigger. A comprehensive overview of the models discussed here is available in an excellent review by Brisse et al (3). Multiple underlying mechanisms have been studied in these models, together demonstrating that aberrant CL activity is likely multifactorial. In this article, we review those studies, considering first the cell intrinsic and extrinsic factors driving excessive CL activation. We also examine the ways in which activated CLs mediate tissue pathology. Finally, we review the mechanisms by which impaired cytotoxicity impedes effective termination of the immune response and restoration of homeostasis.

## 2 Increased antigen presentation produces hyperactive CLs in fHLH

Excessive expansion of antigen-specific CLs is a key feature of fHLH. This finding is recapitulated in the *Prf1^-/-^* model, in which LCMV infection results in increased expansion and persistence of CLs (10, 14), making this a useful system to evaluate several hypotheses that have been proposed to explain this finding. One potentially straightforward explanation for the high levels of CL activation in fHLH is increased antigen burden owing to failed target cell killing. However, Lykens et al. measured viral titers in mice shortly after LCMV infection and demonstrated no differences in viral burden. They went on to show that increased activation also cannot be attributed to intrinsic CL hyperreactivity, as *ex vivo* stimulation of WT and *Prf1^-/-^* CLs resulted in no differences in activation. They did, however, demonstrate that following LCMV infection, IFNγ-producing CD8+ T cells in *Prf1^-/-^* mice had evidence of recent and robust T cell receptor (TCR) signaling. Interestingly, when they co-cultured CD8+ T cells with splenic antigen presenting cells (APCs) from LCMV-infected mice, they found significantly higher levels of IFNγ in the *Prf1^-/-^* culture, suggesting that increased CD8+ T cell activation is due to increased antigen presentation. They did not, however, observe any differences in APC number between WT and *Prf1^-/-^* mice, leading them to conclude that the APCs themselves acquire increased antigen presenting capabilities through mechanisms that remain to be explored (15). These data support the notion that excessive CD8+ T cell activation in fHLH is not directly mediated by the persistence of antigen, but rather, it is due to a central role for the cytotoxicity pathway in immunoregulation.

**Figure 1:**
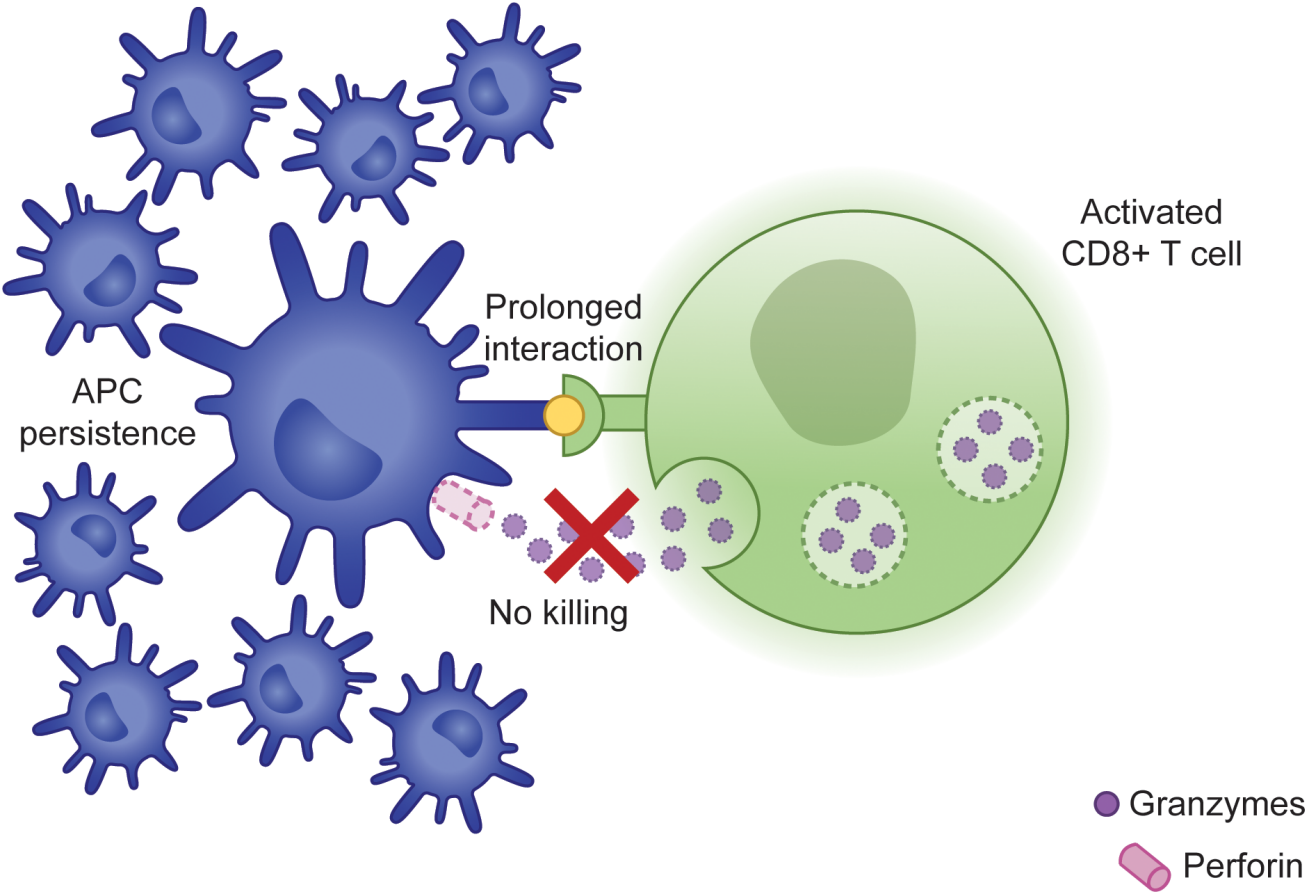
Failure of CLs to induce APC apoptosis results in prolonged synapse time and persistence of APCs capable of promoting further immune activation.

Activation of CD8+ T cells requires the help of APCs, including dendritic cells (DCs) and macrophages. APCs internalize and cleave antigens detected throughout peripheral tissues and load the resulting peptides onto MHC I molecules. Subsequent recognition of these peptide-MHC I complexes by antigen-specific CD8+ T cells facilitates their activation and proliferation (16). In contrast to the work by Lykens et al., other studies have demonstrated that CD8+ T cells play a central role in eliminating antigen-primed DCs, thereby restricting their potential for ongoing antigen presentation (**Figure 1**). When Hermans et al. injected non-antigen-loaded DCs into mice, they could identify these cells in draining lymph nodes several days following injection. Interestingly, the use of antigen-loaded DCs resulted in significantly fewer lymph node DCs, a finding that was reversed following CD8+ T cell depletion, suggesting that antigen-specific CD8+ T cells are responsible for restricting the size of the DC population (17). This same group went on to evaluate the importance of an intact cytotoxicity pathway for this response. To do this, they immunized WT or *Prf1^-/-^* mice with antigen-loaded DCs. When they re-exposed WT mice to unloaded or loaded DCs, only unloaded DCs could be detected in the draining lymph nodes. However, in the *Prf1^-/-^* mice, both unloaded and loaded DCs could be detected, demonstrating that perforin expression is required for elimination of antigen-loaded DCs (18). Specifically, cytotoxicity-competent CD8+ T cells restrict the size of the DC population by inducing apoptosis. When WT LCMV-infected mice are treated with a pan-caspase inhibitor, DC-mediated antigen presentation is increased to levels comparable to those seen in *Prf1^-/-^* mice, while administration of the inhibitor to *Prf1^-/-^* mice does not further augment DC function (19).

This DC persistence in the context of impaired cytotoxicity is functionally important to the magnitude of the resulting immune response. For example, it has been shown that selective depletion of DCs in LCMV-infected *Prf1^-/-^* mice decreases IFNγ production (19). Chen et al. further evaluated the significance of this mechanism using *Prf1^-/-^* mice with DC-specific deficiency of Fas, as Fas signaling has been shown to synergize with perforin in mediating DC apoptosis (20). In these mice, they observed a significant expansion of the DC population, concomitant with profoundly higher levels of IFNγ, increased lymphocytic infiltration into organs, more severe pancytopenia, and early lethality, suggesting that persistence of the DC population is directly associated with the clinical manifestations of fHLH (21).

Following APC-mediated activation, antigen-specific CD8+ T cells rapidly proliferate, expanding the capacity for a targeted immune response to a given antigen. These cells then circulate and engage antigen-expressing target cells *via* their TCR, which activates an intracellular signaling cascade leading to the formation of the IS and subsequent target cell killing (22). Analyses of patient samples have demonstrated that, following activation, CD8+ T cells from patients with fHLH display abnormal immunophenotypes. While the mechanisms leading to this altered cell surface marker expression are poorly understood, they may contribute to aberrant behaviors of activated CD8+ T cells in fHLH. For example, multiple studies have demonstrated that patients with fHLH have an expanded population of CD8+ T cells that are deficient in CD5 expression (23–26). CD5 is a cell surface receptor that localizes to the IS following target cell engagement (27) and provides an inhibitory signal to limit the amplitude of TCR signaling (28). In a normal immune response, CD8+ T cells retain CD5 expression following activation. While additional studies are needed to better understand the significance of CD5 downregulation in fHLH, it can be speculated that loss of CD5 expression on activated CD8+ T cells may contribute to their excessive proliferation by allowing for more dynamic signaling downstream of the TCR (29). Other studies have demonstrated that CD8+ T cells from patients with fHLH express higher levels of HLA-DR (26, 30), an MHC II molecule expressed predominantly on APCs. Acquisition of HLA-DR expression on CD8+ T cells results in upregulation of the proteins required for antigen processing and loading, suggesting that these CD8+ T cells may acquire novel antigen presenting capabilities that serve to further amplify the immune response (31). One possible mechanism underlying the acquisition of HLA-DR expression is trogocytosis, the transfer of plasma membrane proteins from one cell to another following cell-cell contact. This process has been shown to be an important immunoregulatory mechanism impacting CD8+ T cell activation (32), and further studies are needed to determine its potential role in fHLH.

In cytotoxicity-competent CLs, target cell killing triggers disassembly of the IS, *de novo* synthesis of lytic proteins, and identification of another target cell, allowing a single CL to mediate serial killing of multiple antigen-expressing cells (33). Using time-lapse microscopy of *in vitro* interactions between CLs and target cells, Jenkins et al. demonstrated that *Prf1^-/-^* CLs displayed normal kinetics of IS formation but delayed target cell detachment relative to WT cells. Interestingly, they observed the same findings using granzyme-deficient CLs, despite no differences in the rate of perforin pore formation or degranulation, suggesting that target cell death itself provides the required signal for CL detachment. In addition to failed target cell elimination allowing for antigen persistence, this prolonged synapse time directly resulted in increased secretion of the CL cytokines IFNγ, interleukin-2 (IL-2), and TNFα (34).

## 3 Activated CLs directly and indirectly mediate tissue damage in fHLH

The CL-derived cytokines IFNγ, IL-2, and TNFα are significantly elevated in patients with fHLH and have been shown to mediate much of the pathology of this disease (35, 36). Consequently, these cytokines and their downstream signal transduction pathways have been the focus of targeted therapeutic strategies to overcome hyperinflammation. IFNγ is produced by many lymphocyte populations. In LCMV-infected *Prf1^-/-^* mice, activated CD8+ T cells are the primary source (10). Interestingly, work by Gather et al. demonstrates that in *Prf1^-/-^* mice infected with murine cytomegalovirus (MCMV) instead of LCMV, CD4+ T cells and NK cells contribute a greater proportion of IFNγ, suggesting that the pattern of IFNγ production may be trigger-dependent (37). The IFNγ receptor (IFNγR) signals *via* JAK1 and JAK2 to activate STAT1, which induces the expression of a wide variety of target genes, known as interferon-stimulated genes (ISGs) (38). While IFNγR is expressed on many tissue types, macrophages are a particularly important target in fHLH, with IFNγ stimulation driving macrophage activation and secretion of the pro-inflammatory cytokine IL-6 (34). These activated macrophages go on to mediate hemophagocytosis, a hallmark feature of fHLH that underlies the consumptive anemia of inflammation (39). Neutralization of IFNγ in LCMV-infected *Prf1^-/-^* mice, but interestingly not MCMV-infected mice (37), has been shown to prolong their survival, concomitant with resolution of anemia (10, 40). In contrast and as expected, animal models have demonstrated that IFNγ depletion does not reduce the number of activated CD8+ T cells or the resulting organ infiltration (40, 41), suggesting that other cytokines contribute importantly to disease pathology.

In contrast to IFNγ, IL-2 is directly responsible for maintenance of the activated CD8+ T cell population. Signaling *via* JAK1 and JAK3 through STAT5, IL-2 plays an important role in lymphocyte differentiation, optimization of immune responses, and restoration of immune homeostasis. In particular, IL-2 activity is required during an immune response to optimize the production of effector CD8+ T cells and to promote differentiation into memory cells capable of responding to future antigen exposure (42). To evaluate the contributions of IL-2 to the pathophysiology of fHLH, Humblet-Baron et al. developed *Prf1^-/-^* mice with selective deletion of CD25, a component of the IL-2 receptor (IL-2R), on CD8+ T cells. Unlike with IFNγ depletion, these mice still developed profound anemia following LCMV infection. However, they had significantly prolonged survival relative to *Prf1^-/-^* mice with a reduction in the size of the activated CD8+ T cell population and an associated decrease in organ infiltration and production of CL cytokines (41). Consistent with this, use of the JAK1/2 inhibitor ruxolitinib has been shown to inhibit IL-2R signaling in CD8+ T cells and to potently induce apoptosis when used in combination with glucocorticoids (43).

While IFNγ and IL-2 are directly responsible for tissue pathology in fHLH, the effect of TNFα neutralization on disease severity and survival of *Prf1^-/-^* mice is less clear (40, 44). However, hypersecretion of TNFα appears to play an important immunomodulatory role. Activation of the TNFα receptor (TNFR) triggers a complex downstream signaling cascade culminating in the activation of the NFκB and AP1 transcription factors, which stimulate upregulation of genes involved in cell proliferation and survival (45). Taylor et al. demonstrated the importance of this pathway to the survival of memory CD8+ T cells. Using the *Prf1^-/-^* model, they evaluated CD8+ T cell responses in naïve mice versus mice previously immunized with LCMV epitopes. Upon re-exposure to LCMV, immunized mice rapidly generated a large number of IFNγ-producing CD8+ T cells, consistent with the presence of a memory CD8+ T cell population. These immunized mice developed more severe disease pathology and had decreased survival relative to naïve mice. Interestingly, they found that immunized mice had a larger subset of TNFα-producing splenic CD8+ T cells and that combined blockade of TNFα and IFNγ more effectively prolonged survival relative to IFNγ blockade alone specifically in immunized mice but not in naïve mice. Together, these data suggest that TNFα plays an IFNγ-independent role in maintaining a memory CD8+ T cell population that contributes to hyperinflammation following repeated or persistent antigen exposure (46).

**Figure 2:**
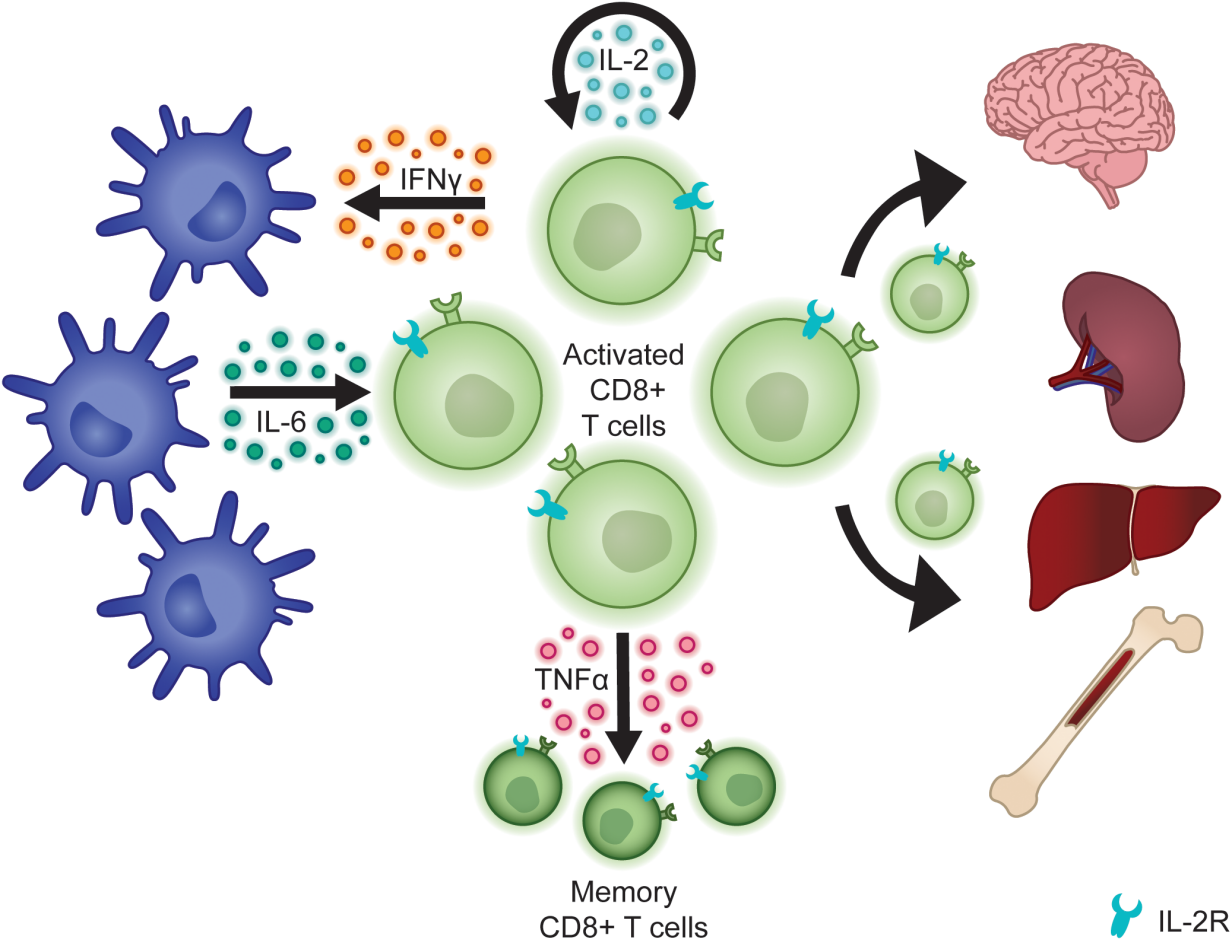
Activated CLs secrete cytokines that sustain the CL population, activate other cells of the innate and adaptive immune system, and promote the development of memory CD8+ T cells. Activated CLs also directly infiltrate tissues to mediate organ damage.

Finally, while activated CLs indirectly mediate tissue damage through the release of inflammatory cytokines, they also directly infiltrate tissues, driving common clinical features of fHLH including hepatosplenomegaly and neurologic dysfunction (47) (**Figure 2**). This has been studied in detail using *Prf1^-/-^* mice, where LCMV infection has been shown to cause increased lymphocytic infiltration of lymph nodes, bone marrow, spleen, and liver, resulting in disrupted tissue architecture (10, 40). Interestingly, Chaturvedi et al. demonstrated that tissue-infiltrating lymphocytes may produce even higher levels of IFNγ than circulating lymphocytes. They analyzed patient T cell phenotypes in peripheral blood versus tissues including bone marrow, cerebrospinal fluid, and lymph nodes. Approximately 80% of these tissue-associated CD8+ T cells were positive for HLA-DR, consistent with a high degree of activation, and were more likely to be IFNγ-producing than were peripheral blood CD8+ T cells (26). Overall, these data suggest that tissue-infiltrating lymphocytes may contribute to disease both by directly mediating tissue damage and by serving as a source for further cytokine secretion.

## 4 Defective cytotoxicity impairs restoration of immune homeostasis

As described above, extensive evidence points to CL hyperactivation as a key driver of the pathophysiology of fHLH, both through the direct actions of CLs themselves and through their ability to activate other immune cell subtypes, which in turn secrete their own inflammatory cytokines and mediate tissue damage. An additional body of work has highlighted several mechanisms through which deficiencies in cytotoxicity contribute to impaired termination of the immune response and failure to restore immune homeostasis. Specifically, in a normal immune response, eradication of the triggering antigen leads to apoptosis of most of the antigen-specific effector cells, providing a safeguard against ongoing inflammation (48). Failure to eliminate these cells, as occurs in the setting of defective cytotoxicity, perpetuates the cycle of hyperinflammation.

NK cells have been shown to play an important role in limiting the size of CD8+ T cell populations. When Waggoner et al. depleted NK cells in WT mice and infected them with LCMV, they observed increased mortality relative to mice with a normal NK cell population, and this was associated with increased expansion of LCMV-specific CD8+ T cells (49). Sepulveda et al. went on to generate mice with targeted perforin deficiency in CD8+ T cells alone versus in both CD8+ T cells and NK cells. Following adoptive transfer of CD8+ T cells into these mice, they observed significantly higher numbers of transferred CD8+ T cells in mice with defective cytotoxicity in both CD8+ T cells and NK cells, relative to CD8+ T cells alone. This experiment further confirms that NK cells play an important role in restricting the size of the CD8+ T cell population and that this activity is dependent on an intact cytotoxicity pathway (50).

Several mechanisms have been proposed to explain how NK cells modulate the size of the CD8+ T cell population. Evidence suggests that this is accomplished at least in part through NK-induced apoptosis of DC cells. During an immune response, a subset of DCs becomes activated by antigen exposure, resulting in maturation that renders them resistant to the cytotoxic effects of NK cells. In contrast, immature DCs remain susceptible to NK-mediated cytotoxicity. By inducing cell death in these immature DCs, NK cells edit the DC population, retaining only those that are most capable of facilitating an immune response (51). This in turn limits the size of the DC population, which, as described above, is important for modulating CD8+ T cell activation and subsequent cytokine release. Marcenaro et al. have demonstrated impairment of this process in patients with fHLH. In their work, they showed that NK cells from healthy donors effectively induce apoptosis of immature allogeneic DCs *in vitro*, while cells from patients with *UNC13D* deficiency do not, confirming that this NK cell function is dependent on intact cytotoxicity (52). In addition to their effect on DCs, NK cells modulate CD8+ T cell activity by restricting CD4+ T cells, which normally provide CD8+ T cell help that optimizes the production of effector and memory cell populations (53). Intriguingly, Waggoner et al. showed that LCMV infection of mice with concomitant depletion of NK cells and CD4+ T cells prevents the expansion of the CD8+ T cell population observed with NK cell depletion alone, suggesting that CD4+ T cell help is important for sustaining this expansion. Furthermore, transfer of splenocytes from LCMV infected mice into control mice resulted in rapid loss of CD4+ T cells, an effect that was overcome with NK cell depletion. This suggests that NK cells restrict CD8+ T cells indirectly through CD4+ T cell-induced apoptosis, which impedes CD4+ T cell help to CD8+ T cells (49). Finally, NK cells may act directly on CD8+ T cells to inhibit their proliferation. Sierra et al. have shown that co-culture of CD8+ T cells with tumor-infiltrating NK cells results in decreased CD8+ T cell proliferation relative to co-culture with naïve NK cells (54), suggesting that activated NK cells act directly on CD8+ T cells to restrict the amplitude of an immune response. Further studies are needed to evaluate this direct effect of NK cells on CD8+ T cells in the context of defective cytotoxicity.

Also important to the maintenance of immune homeostasis are regulatory T cells (Tregs), a subtype of CD4+ T cells that exert immunosuppressive effects *via* release of anti-inflammatory cytokines and cell contact-dependent inhibition of cell proliferation and survival (55). As with CLs, intact cytotoxicity is required for Treg activity. Grossman et al. demonstrated that inhibition of perforin prevents Treg-induced apoptosis of CD8+ T cells and DCs (56), suggesting a direct mechanism by which defective cytotoxicity impairs Treg function. Interestingly, additional studies have demonstrated that Treg dysfunction in fHLH may also be indirectly mediated by competition for IL-2, which is consumed by and required for the maintenance of both effector T cells and Tregs (42). When Humblet-Baron et al. examined Tregs in LCMV-infected mice, they found a significant reduction in the size of the Treg population in *Prf1^-/-^* mice relative to WT mice. The Tregs from *Prf1^-/-^* mice also had decreased cell surface expression of CD25, a surrogate marker of IL-2 utilization (57). In a normal immune response, higher levels of CD25 expression on Treg cells relative to effector cells results in a hierarchy of utilization, whereby Tregs preferentially utilize IL-2 (58). In the *Prf1^-/-^* mice, this hierarchy was inverted, with increased CD25 expression on CD8+ T cells. Furthermore, selective deletion of CD25 expression on CD8+ T cells rescues the size of the Treg population in *Prf1^-/-^* mice (57). Together, these findings suggest that excessive CD8+ T cell activation in fHLH allows these cells to serve as a sink for IL-2, depriving Tregs of this necessary survival factor and restricting their ability to successfully suppress the immune response.

## 5 Concluding remarks

Inherited defects in the lymphocyte cytotoxicity pathway contribute to immune dysregulation through complex mechanisms that confer widespread consequences throughout the immune system. Focusing largely on murine models of fHLH, we demonstrate how hyperinflammation can result from excessive effector cell activation and the inability of these cells to effectively downregulate and/or terminate the immune response. Together, these properties lead to a positive feedback loop that drives sustained immune cell activation, cytokine secretion, and tissue damage. While the treatment of inflammation in fHLH has historically hinged on the use of genotoxic agents, understanding this pathophysiology at the cellular level creates opportunities for further development of rationally designed therapies targeting critical points in this cycle. Many such therapies have already shown promise in patients with fHLH and related immune dysregulation syndromes (59). Ongoing research into these mechanisms of immune dysfunction will support further clinical trials aimed at improving outcomes for patients with fHLH and related disorders of the immune system.

## Author contributions

ES and LM wrote the manuscript. KN reviewed and edited the manuscript. All authors contributed to the article and approved the submitted version.
